# O-mannosylation and furin processing are critical maturation events for the KIAA1549 protein implicated in pediatric pilocytic astrocytomas

**DOI:** 10.1016/j.jbc.2026.113403

**Published:** 2026-08-07

**Authors:** Gloria Kyrila, Serpil Ahmed, Anna Borgenvik, Daniel Christen, Sean A. Misek, Alexander Zhang, Sanae Furukawa, Hiren J. Joshi, Yoshiki Narimatsu, Rameen Beroukhim, Pratiti Bandopadhayay, Adnan Halim

**Affiliations:** 1 Copenhagen Center for Glycomics & Center for Glycocalyx Research, Department of Cellular and Molecular Medicine, University of Copenhagen, Copenhagen, Denmark; 2 Dana-Farber/ Boston Children's Cancer and Blood Disorders Center, Boston, Massachusetts, USA; 3 Broad Institute of MIT and Harvard, Cambridge, Massachusetts, USA; 4 Department of Pediatrics, Harvard Medical School, Boston, Massachusetts, USA; 5 Institute of Molecular Medicine, ZBMZ, Faculty of Medicine, University of Freiburg, Freiburg, Germany; 6 Department of Cancer Biology, Dana-Farber Cancer Institute, Boston, Massachusetts, USA; 7 Department of Medical Oncology, Dana-Farber Cancer Institute, Boston, Massachusetts, USA; 8 Department of Medicine, Harvard Medical School, Boston, Massachusetts, USA

**Keywords:** KIAA1549, KIAA1549::BRAF fusion, O-mannosylation, protein maturation, POMT1/POMT2, furin processing, ER-Golgi trafficking, protein glycosylation, pediatric pilocytic astrocytoma, oncogenic signaling

## Abstract

The gene fusion KIAA1549::BRAF is a common oncogenic driver in pediatric pilocytic astrocytoma. The chimeric fusion protein that comprises most of the type 1 transmembrane KIAA1549 protein fused in its C-terminal cytosolic domain to the truncated BRAF oncogene without its N-terminal autoinhibitory domains. KIAA1549 has a large N-terminal ectodomain that undergoes extensive O-mannosylation directed by the POMT1/2 protein O-mannosyltransferase complex localized in the ER. Here, we investigated how posttranslational modifications contribute to KIAA1549 protein maturation in the secretory pathway. Using a panel of KIAA1549 expression constructs and glycoengineered HEK293 cells, we provide evidence that O-mannosylation of the KIAA1549 ectodomain is required for ER exit, acquisition of complex N-glycans, and subsequent furin-like cleavage in the Golgi. In the absence of POMT1/2 O-mannosylation KIAA1549 is retained in ER and fails to undergo proprotein processing. Our findings confirm and extend our previous studies with the KIAA1549::BRAF fusion protein, where oncogenic transformation was dependent on POMT1/2 O-mannosylation and affected on furin processing. Thus, these intrinsic posttranslational modifications of the KIAA1549 ectodomain are critical determinants of the cellular trafficking that lead to activation of the BRAF oncogene, potentially involving a cascade of cleavage events in the Golgi that ultimately release a truncated but fully active BRAF kinase domain into the cytosol.

While the function of wild-type KIAA1549 remains largely unexplored, the gene is widely implicated in pediatric low-grade gliomas (1). KIAA1549::BRAF (K::B) fusions represent a hallmark genetic alteration of pilocytic astrocytoma, the most common brain tumor in children (2, 3). The most frequent rearrangement arises from a tandem duplication that joins exon 15 or 16 of KIAA1549 to exon 9 or 11 of BRAF (4), resulting in chimeric KIAA1549::BRAF fusions composed of nearly the entire KIAA1549 coding region with its ectodomain, transmembrane segment (TM), and a truncated cytoplasmic domain, fused in-frame to the BRAF kinase domain without the CR1 and CR2 autoinhibitory modules (Fig. 1*A*) (5, 6). The full coding region of KIAA1549 predicts a type I transmembrane protein, and we originally identified extensive O-linked mannose (O-Man) glycosylation in the N-terminal region of the ectodomain (7) and recently proposed that O-Man is necessary for oncogenic signaling and tumorigenesis (6). We found that the KIAA1549::BRAF-driven oncogenic transformation was dependent on ER-localized POMT1/2 mannosyltransferases and O-mannosylation of the KIAA1549 ectodomain before maturation by proprotein processing within the ectodomain by a furin-like protease (6). These findings suggest that O-mannosylation of the KIAA1549 component of the KIAA1549::BRAF fusion protein and its furin processing are critical for activation of the BRAF proto-oncogene.

**Figure 1:**
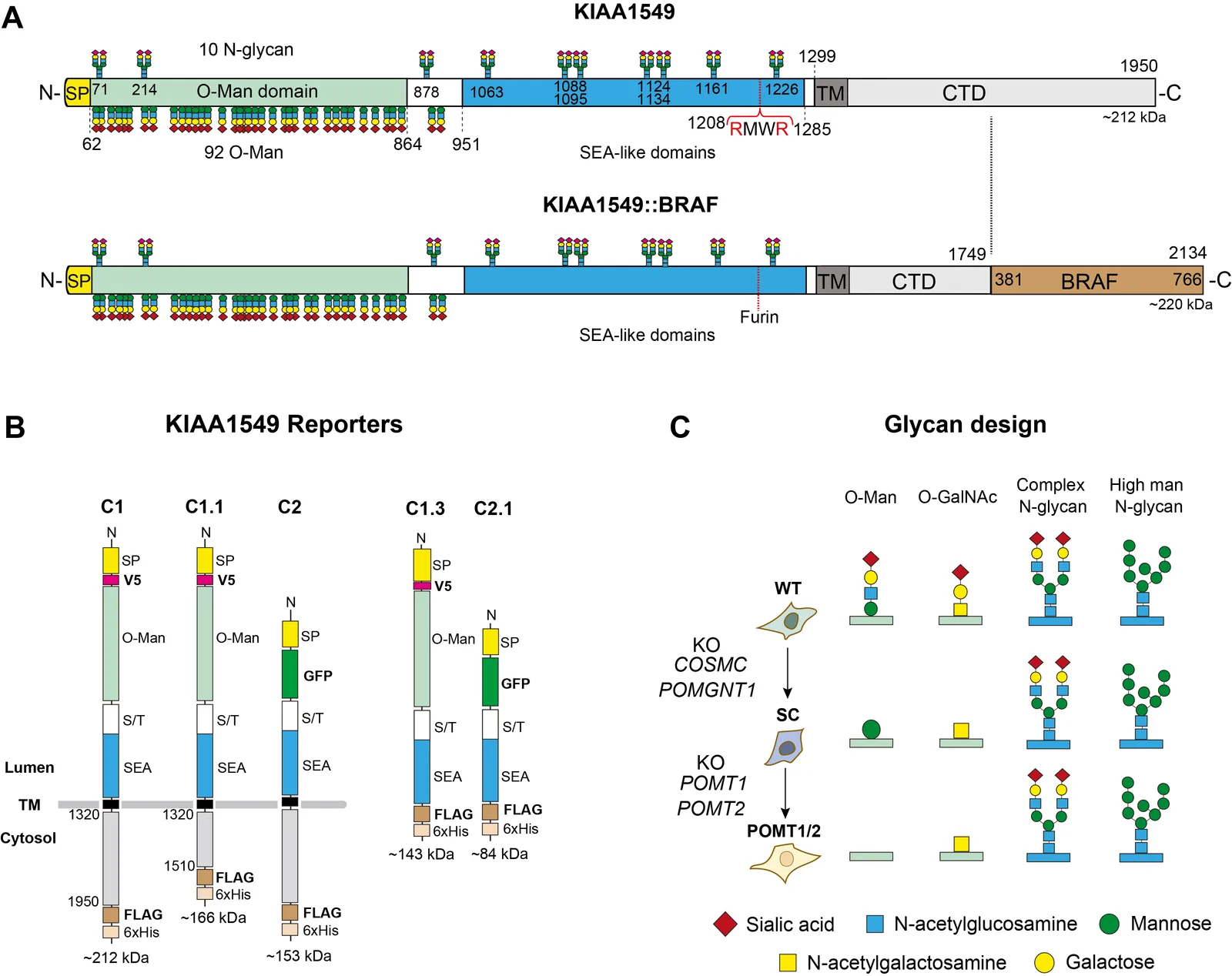
**Overview of KIAA1549 expression constructs and genetic glycoengineering employed.***A*, graphic depiction of the full coding KIAA1549 WT protein (*top*) and the KIAA1549::BRAF fusion protein (*bottom*) with location of 10 predicted N-glycans and the O-mannosylated domain (O-Man) with at least 92 identified O-Man glycans (7, 8, 9). The WT KIAA1549 encodes a protein with a 61 amino acid N-terminal signal peptide (SP), O-Man domain (aa 62-864), a Ser/Thr-rich region (aa 865-950), three predicted SEA-like domains (aa 951-1285) including the putative furin cleavage site (R^1208^MWR^1211^), transmembrane region (TM), and C-terminal domain (CTD). The most common KIAA1549::BRAF 15/9 fusion truncates the CTD of KIAA1549 (aa 1749) with the in-frame fused BRAF kinase (aa 381-766). Note that the Ser/Thr-rich region (aa 865-950) may represent an extension of the O-Man domain given its similar sequence features, but we excluded this from the O-Man domain analysis in our study as we have not identified O-Man glycans in this segment. This could, however, be due to lack of appropriate trypsin digestion motifs. *B*, graphic depiction of the from KIAA1549 expression constructs used with indications of inserted antibody tags (V5, FLAG, 6×His) and the swapping of the O-Man domain with GFP. Approximate molecular weight (MW) of proteins without glycans are indicated. *C*, depiction of the genetic glycoengineering dissection strategy in HEK293 cells used for expression of KIAA1549 variants and the predicted glycosylation outcome. HEK293^WT^, HEK293^SC^ (KO:COSMC/POMGNT1) with truncated O-Man and O-GalNAc glycans, and HEK293^SC/POMT1/2^ (KO:COSMC/POMGNT1/POMT1/2) with additional loss of POMT1/2 O-mannosylation capacity we used. The predicted glycans attached to KIAA1549 ectodomain are illustrated with glycan symbols (SNFG annotation). Representative N-glycan structures for high-Man and complex type N-glycans are shown.

We previously identified KIAA1549 in a screen for O-Man glycosylated proteins using the SimpleCell O-glycoproteomics strategy that employs genetically engineered cells without capacity for elongating O-glycans (7, 8, 9). KIAA1549 contains an extended N-terminal ectodomain (aa 62-864) in which we identified >70 O-Man glycans introduced by the POMT1/2 protein O-mannosyltransferases (7). Interestingly, POMT1/2 only serve a very limited number of proteins with α-dystroglycan and KIAA1549 being among the most representative protein substrates (8), which contain distinct domains with multiple O-Man glycans that are elongated (8, 10). Most other O-mannosylated proteins, including the larger cadherin and plexin superfamilies, are O-mannosylated within distinct protein folds (Ig-like domains) by distantly related TMTC1-4 and TMEM260 protein O-mannosyltransferases in the ER (11, 12). The O-Man glycodomain (aa 72-864) of KIAA1549 is reminiscent of the mucin-like domain found in α-dystroglycan, which contains an N-terminal region densely modified by O-Man glycans (N-terminal half) and a C-terminal region densely decorated by GalNAc-type O-glycans (C-terminal half); however, unlike α-dystroglycan, GalNAc-type O-glycosylation has not been reported on KIAA1549. While most of the O-Man glycans are elongated by the POMGNT1 β2GlcNAc-transferase, select O-Man glycans in α-dystroglycan are elongated by the POMGNT2 β4GlcNAc-transferase (M3 core) to form the matriglycan polysaccharide that mediates interactions with laminin and other extracellular matrix components (10). The O-Man glycodomain in KIAA1549 is longer and represents the most heavily O-mannosylated mammalian protein known to date. However, there is currently no evidence to suggest that the KIAA1549 O-Man glycans are extended with the M3 core structure and matriglycan. The nature and functions of the KIAA1549 protein and its post-translational modifications remain unexplored.

Here, we aimed to shed light on the KIAA1549 protein and the glycosylation and proprotein processing that serve the KIAA1549::BRAF fusion protein and oncogenic transformation (6). We employed a series of KIAA1549 expression designs and genetic dissection of the glycosylation capacities in HEK293 cells (Fig. 1) to demonstrate that POMT1/2 O-mannosylation of the ectodomain is required for exit from ER and acquisition of complex-type N-glycans. Moreover, we demonstrate by pharmacologic inhibition that the KIAA1549 protein upon ER exit undergo efficient furin-like proprotein processing and secretion of the ectodomain as well as further cleavage in the cytosolic region. Our study provides insights into the molecular requirements that govern maturation of the KIAA1549 protein and how its post-translational modifications in ER and Golgi govern the oncogenic activity of the KIAA1549::BRAF fusion.

## Results

### Construct design and glycoengineering strategy to dissect O-Man functions on KIAA1549

Inspired by our studies of the KIAA1549::BRAF fusion protein (6), we designed a series of expression constructs based on the full coding KIAA1549 WT protein to investigate roles of the N-terminal O-mannosylated domain and other structural features, including a furin proprotein convertase cleavage site (RMWR^1211^), the ectodomain with ten predicted N-glycans, and the transmembrane domain (^1299^TM^1319^) (Fig. 1, *A* and *B*). Our construct designs included N-/C-terminal-tagged variants of full-coding, C-terminal-truncated, and secreted ectodomain reporters, together with variants in which the O-Man domain was replaced with a GFP module (see Fig. S1 for sequence information).

We previously demonstrated that O-mannosylation of KIAA1549 is solely dependent on the POMT1/2 O-mannosyltransferase complex (8, 9). To probe the role of O-mannosylation in KIAA1549, we employed our genetic dissection strategy, combining knockout (KO) of elongation enzymes and POMT1/2, as illustrated in Figure 1*C*. We used the SimpleCell (SC) strategy with combinatorial KO of COSMC (blocking elongation of O-GalNAc glycans) and POMGNT1 (blocking elongation of O-mannose glycans), to truncate both O-glycan types and simplify analysis, as previously described (7, 13). HEK293^WT^ cells produce elongated O-Man glycans (mainly NeuAc-Gal-GlcNAc-Manα1-O-Ser/Thr) and O-GalNAc glycans (mainly NeuAc-Gal-GalNAcα1-O-Ser/Thr), while the corresponding HEK293^KO:COSMC/POMGNT1^ cell (HEK^SC^) generates only the truncated monosaccharide forms (Manα1-O-Ser/Thr and GalNAcα1-O-Ser/Thr) (Fig. 1*C*). We used the HEK^SC^ cell background to engineer cells lacking POMT1/2-dependent O-mannosylation (HEK^SC/KO:POMT1/2^) to probe effects of O-mannosylation of KIAA1549, since we previously found that elongation of O-Man glycans did not alter the protein processing and oncogenic properties of the KIAA1549::BRAF fusion protein (6).

### O-mannosylation controls access to Golgi and furin-mediated cleavage of KIAA1549

**Figure 2:**
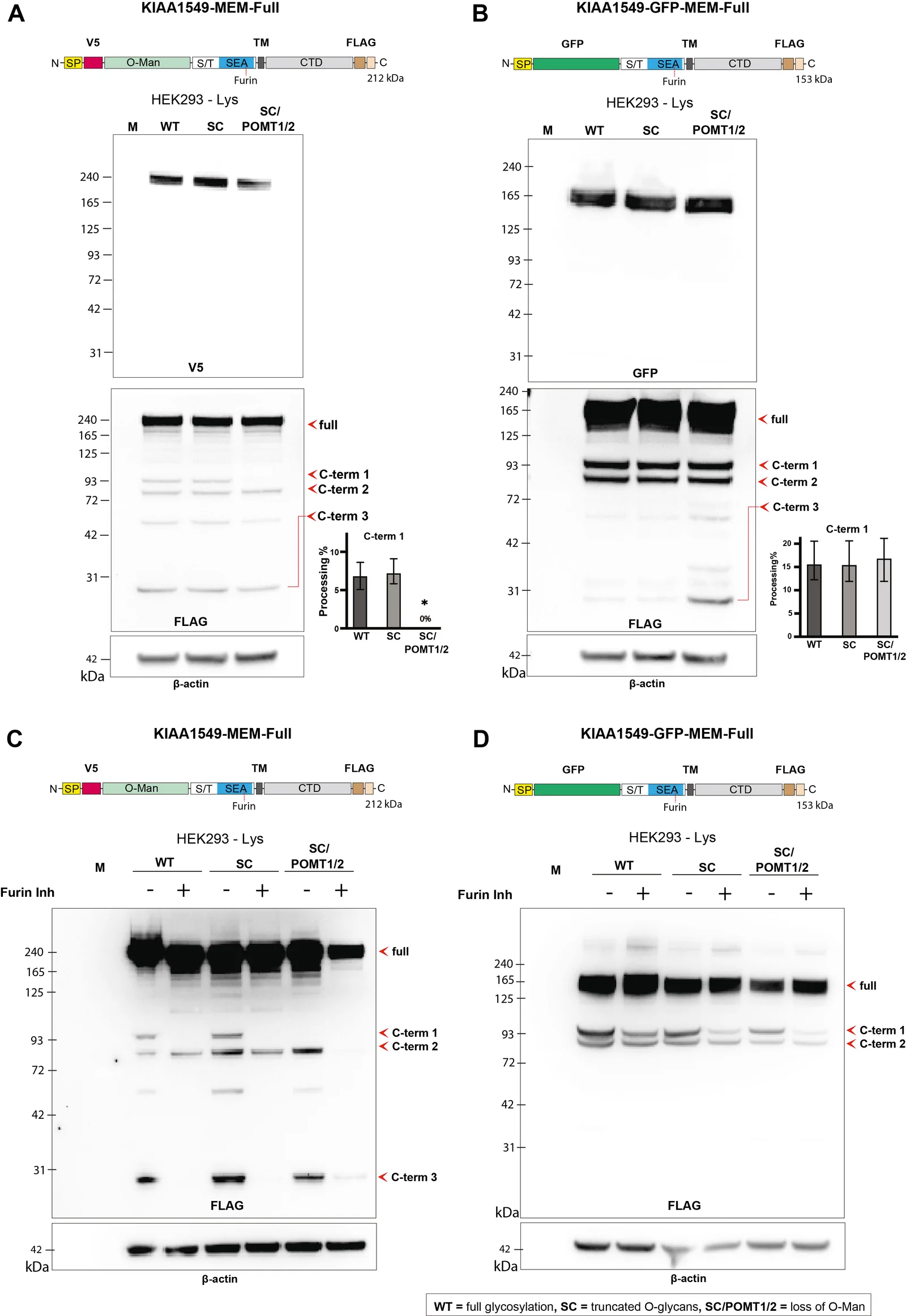
**The KIAA1549 protein requires POMT1/2 O-mannosylation to access Golgi and undergo proprotein convertase processing.***A*, Western blot analyses (V5/FLAG) of total lysates of HEK293 glycoengineered cells transiently expressing the full coding KIAA1549-MEM-Full. The predicted intact protein (240 kDa) and three minor C-terminal fragments (C-term #1-3) are indicated. Note that C-term #1 is not observed when expressed in HEK293^SC/POMT1/2^ (*right insert panel* shows band quantification, ANOVA *p* = 0.025, n = 3; mean ± SD). *B*, the same analysis as (*A*) with cells transiently expressing the KIAA1549-GFP-MEM-Full swapped protein. *C*, Western blot analysis of total lysates of HEK293 glycoengineered cells transiently expressing KIAA1549-MEM-Full and incubated for 48 h with/without the dec-RVKR-CMK furin inhibitor I (50 μm). Note that furin inhibition abolished the appearance of the C-term #1 as well as C-term #3 bands, whereas C-term #2 was unaffected. *D*, The same analysis as (*C*) with cells transiently expressing the KIAA1549-GFP-MEM-Full swapped protein. Note that furin inhibition partly abrogated appearance of the C-term #1 band, whereas C-term #2 was unaffected.

We experienced challenges with obtaining stable expression of the KIAA1549 constructs in the panel of glycoengineered HEK293 cells, and we therefore had to resort to transient expression for screening. We first transiently expressed the full coding KIAA1549 gene (KIAA1549-MEM-Full) in our panel of glycoengineered HEK293 cells and analyzed total cell lysates by Western blot analysis using N- and C-terminal tags for detection (Fig. 2*A*). The predicted full protein migrating around 240 kDa was found as the major band in lysates of all engineered cells. Surprisingly, only a minor fraction of the protein was processed into the predicted furin-cleaved products, which we previously observed as the major form of the KIAA1549::BRAF fusion protein when expressed stably in h9 human embryonic neural stem cells (h9-NSC) (6). Three FLAG-reactive bands designated C-term #1 and #3 migrating around ∼100 kDa and ∼30 kDa, respectively, were observed. However, as expected from our analysis of KIAA1549::BRAF, the predicted furin-cleaved product (C-term #1, corresponding to the full C-terminal fragment ∼100 kDa) was detected only upon expression in HEK^WT^ and HEK^SC^, but not in HEK^SC/KO:POMT1/2^ cells. Although the C-term #1 was a minor band, this result indicates that POMT-mediated O-mannosylation in ER is required to generate this protein fragment. Notably, the C-term #2 and #3 fragments were generated independent of POMT1/2-mediated O-mannosylation, indicating that the cytosolic C-terminal domain may undergo other processing as well, at least when transiently overexpressed as performed in this experiment.

We then expressed the KIAA1549-GFP-MEM-Full construct, in which the O-Man domain (aa 72-864) was replaced by a GFP module, and confirmed expression of the predicted full coding protein by Western blot analysis (note that the expression levels estimated by anti-FLAG was higher than observed with the KIAA1549-MEM-Full construct) (Fig. 2*B*). The KIAA1549-GFP-MEM-Full protein migrated at the predicted mass (165 kDa) and was detectable as the major band in lysates of all engineered cells (Fig. 2*B*). Two smaller fragments corresponding to the C-term #1 and #2 fragments were also prominent in all engineered cells, including in HEK^SC/KO:POMT1/2^ without POMT1/2 O-mannosylation capacity. A lower intensity band corresponding to C-term #3 was also detected, but variably and hardly above background. This suggests that it is the O-Man domain that requires O-mannosylation for reaching the Golgi, and subsequently for efficient furin cleavage. To further confirm the involvement of furin-mediated processing, we treated cells with the furin inhibitor dec-RVKR-CMK. Furin inhibition abolished cleavage of the KIAA1549-MEM-Full protein into the C-term #1 fragment only found in cells with POMT1/2 O-mannosylation capacity (Fig. 2*C*). Interestingly, fragments C-term #2 and #3 were observed in all three engineered cells, although with varying intensities (Fig. 2*A*). The C-term #2 fragment was present independent of furin inhibition in HEK^WT^ and HEK^SC^, but lost in the *POMT1*/*2* KO background with furin inhibition (Fig. 2*C*). The intensity of the smallest C-term #3 fragment was substantially reduced with furin inhibition in *POMT1*/*2* KO cells. Since the C-term #1 furin sensitive fragment was not detectable in lysates of HEK^SC/KO:POMT1/2^ cells, the origin of the C-term #2 and #3 fragments is difficult to determine. However, comparing these fragment banding patterns with the fragments found for the KIAA1549-GFP-MEM-Full domain-swapped protein with the same furin site and C-terminal sequence (Fig. 2*D*), we note that only the C-term #1 and #2 fragments are reproducibly detectable.

We next performed the same furin inhibition analysis with the KIAA1549-GFP-MEM-Full construct (Fig. 2*D*), which similarly showed that cleavage into the C-term #1 fragment was inhibited, although to a lesser degree than observed for the WT KIAA1549 protein. The reason for the relatively lower cleavage efficiency is unclear as the predicted furin sites are identical, but the relative higher expression of the KIAA1549-GFP-MEM-Full protein and its secretion to Golgi may account for higher substrate availability and unfavorable conditions for inhibition. Note, that the KIAA1549-GFP-MEM-Full construct mainly produced detectable C-term #half fragments and further visible degradation products were only found in lysates from cells without POMT1/2 O-mannosylation capacity (Fig. 2*B*). The C-term #3 band was not readily detected with the KIAA1549-GFP-MEM-Full construct, although a visible band was apparent with variable intensity in experiment when expressed in HEK^SC/KO:POMT1/2^ (Fig. 2*B*).

These results demonstrate that KIAA1549 requires POMT1/2 O-mannosylation within the O-Man domain (aa 72-864) to undergo furin-mediated cleavage in the juxtamembrane region, presumably by reaching the Golgi where furin processing is known to occur (14).

### Transient expression of KIAA1549 results in accumulation of immature proteoforms presumably in ER, with only a minor fraction reaching the Golgi

When expressing the KIAA1549-MEM-Full and KIAA1549-GFP-MEM-Full constructs transiently we primarily detected these as intact proteins migrating at their predicted molecular weights of ∼240 kDa and ∼165 kDa, respectively, suggesting that most of the KIAA1549 protein may be retained in the ER when transiently overexpressed in HEK293 cells irrespective of the O-mannosylation capacity (Fig. 2). In contrast, we previously observed that stable expression of KIAA1549::BRAF in h9 NSCs cell pools through viral transduction predominantly resulted in the processed (furin-cleaved) form (6). To resolve this discrepancy, we first directly compared transient transfection and viral transduction of KIAA1549::BRAF in HEK293 cells and observed that the much lower expression levels found with viral transduction resulted predominantly in the furin-processed form of the fusion protein (Fig. S2), similar to that seen in h9 NSCs. These observations suggest that the expression levels may influence KIAA1549 trafficking and processing. Despite multiple attempts, we were unable to clone and establish glycoengineered HEK293 cells with stable expression following antibiotic selection and the virally transduced HEK293 cells rapidly lost detectable expression (not shown). Nevertheless, these results suggest that the overexpression exceeds the export capacity of the ER, leading to severe ER stress and accumulation of KIAA1549 in the cells.

To further probe localization of the intact and processed KIAA1549 protein fragments, we took advantage of the predicted N-glycosylation of the ectodomain with a total of nine N-glycans positioned N-terminal of the putative furin processing site (R^1208^MWR^1211^) and one N-glycan C-terminal of this site (Fig. 1*A*). We used sensitivity to Endo H cleavage of high-Man N-glycans as a surrogate for ER localization and PNGase F for release of both high-Man and complex N-glycans to determine the full range of potential gel mobility shifts. Although complete Endo H sensitivity is generally consistent with ER localization, some glycoproteins can traffic through the Golgi while retaining unprocessed high-Man N-glycans. In addition, glycan maturation may be limited by site accessibility, and overexpression-induced ER stress or disruption of the secretory pathway can impair normal glycoprotein processing and secretion. Therefore, our findings should be interpreted within these limitations, and further studies will be required to determine the subcellular localization and trafficking of KIAA1549.

**Figure 3:**
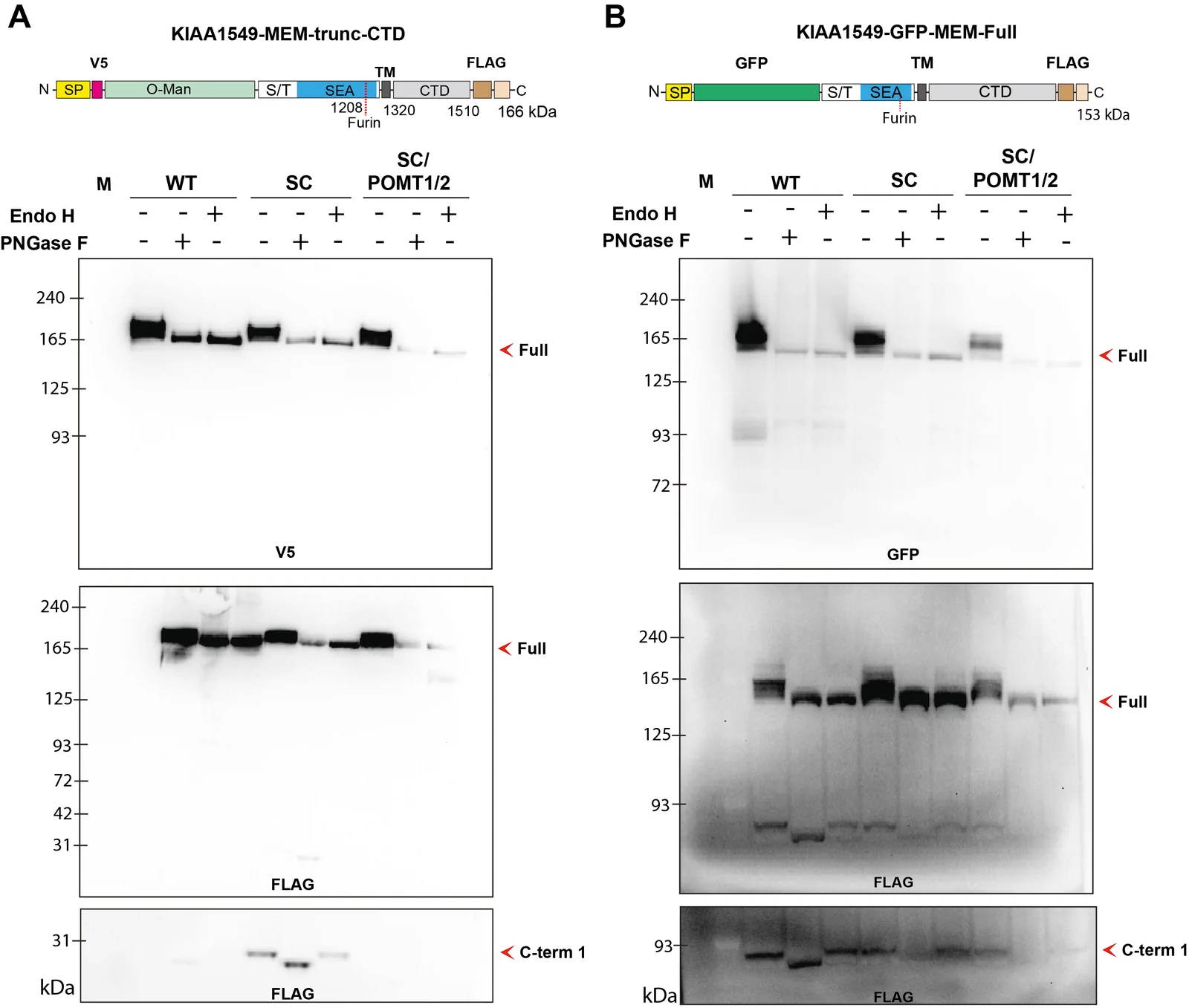
**N-glycan analysis of the intact KIAA1549 protein is consistent with ER localization and that the minor furin-cleaved C-terminal fragment reaches the Golgi.***A*, Western blot analysis of total lysates of HEK293 glycoengineered cells transiently expressing the C-terminal truncated KIAA1549-MEM-CTDtrunc and subjected to Endo H or PNGase F treatment as indicated. Note, the same mobility shifts for the intact protein (now migrating at ∼165 kDa) were observed for both Endo H and PNGase F treatment, indicating the presence of high-mannose N-glycans. *B*, analysis as in (*A*) with the KIAA1549-GFP-MEM-Full construct showing the same sensitivity for the intact protein. Note, that the predicted furin-processed band (C-term #1, ∼80 kDa) was Endo H resistant, indicating the presence of complex-type N-glycans and further maturation in the Golgi.

We first analyzed the KIAA1549-MEM-Full construct without being able to resolve mobility shifts upon removal of N-glycans by SDS-PAGE analysis (not shown). We therefore included a C-terminal truncated KIAA1549-MEM-CTDtrunc construct (MW: ∼166 kDa) to express in our panel of glycoengineered HEK293 cells, which showed a minor change in mobility after digestion with both Endo H and PNGase F with all cells (Fig. 3*A*). The same analysis with the smaller GFP-substituted KIAA1549-GFP-MEM-Full protein (MW:∼153 kDa) recapitulated this result but with more clear separation (Fig. 3*B*). These results show that the KIAA1549 protein variants regardless of O-mannosylation are equally sensitive to Endo H and PNGase F and thus predicted to only contain high-man N-glycans. This finding suggests that the mature KIAA1549 proteins have not reached the Golgi for complex N-glycosylation and hence are predicted to accumulate in the ER. In contrast, focusing on the minor furin-processed C-term #1 fragment revealed that this was insensitive to Endo H but sensitive to PNGase F digestion, demonstrating that this fragment with a single N-glycan is derived from KIAA1549 with complex N-glycans. These results suggest that KIAA1549, when transiently overexpressed, is accumulated in cells, likely in ER, and that the minor amount that reaches Golgi undergoes complex N-glycosylation and full furin cleavage.

### Secretion of the KIAA1549 ectodomain

The complete furin processing of the minor part of the KIAA1549 protein that exits the ER may suggest that the ectodomain is secreted to the surface or to the culture medium. We were unable to detect shed ectodomains in culture media by Western blot analysis from either KIAA1549-MEM-Full (using N-terminal V5) or KIAA1549-GFP-MEM-Full (using GFP) transiently transfected cells even after concentration, and we also did not detect significant surface expression (not shown).

**Figure 4:**
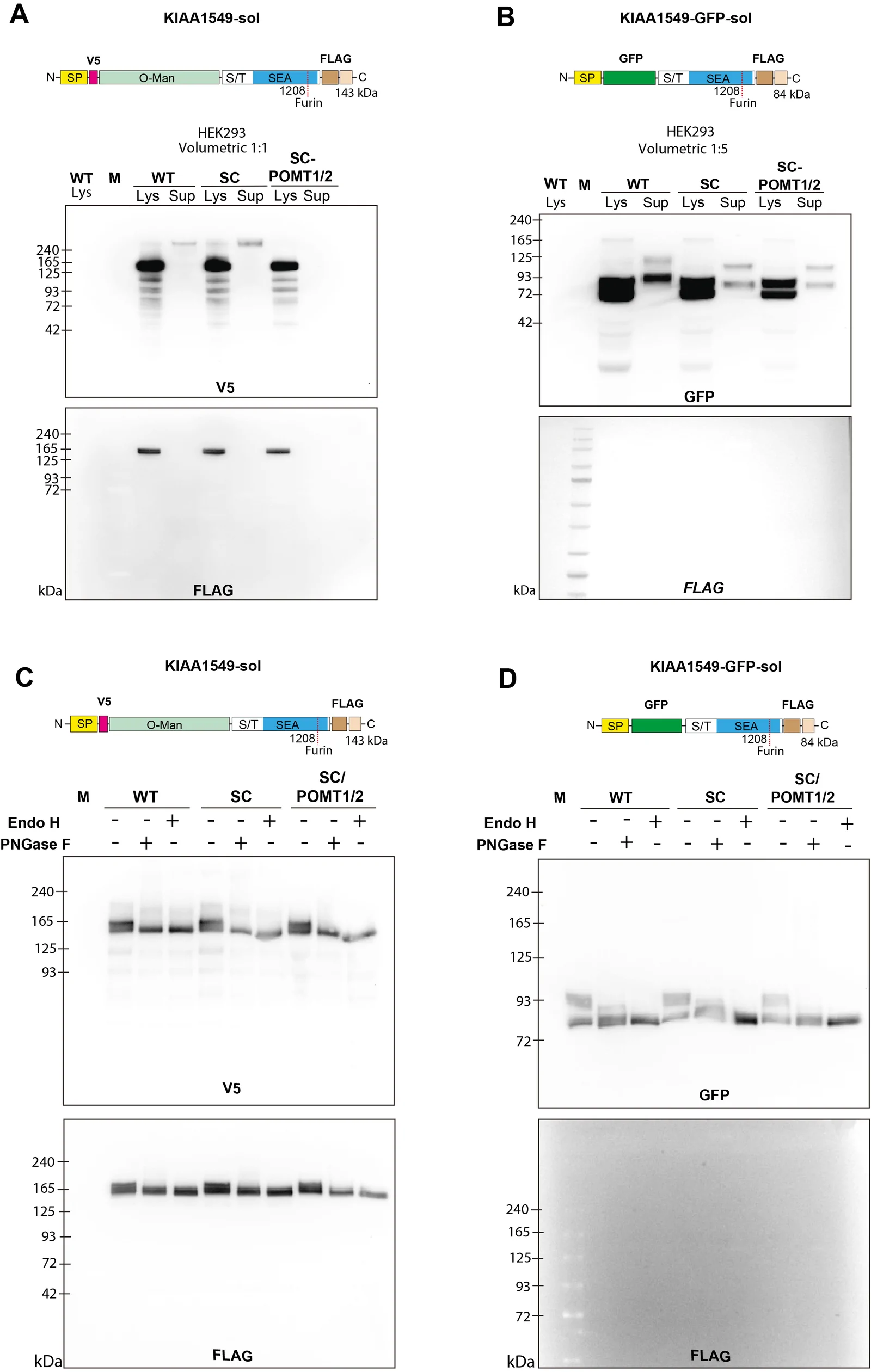
**The soluble ectodomain of KIAA1549 requires O-mannosylation for ER exit and secretion.***A*, Western blot analysis of cell lysates (Lys) and culture medium (Sup) of glycoengineered HEK293 cells transiently expressing the secreted KIAA1549-sol construct. Note, that a secreted V5-labelled and FLAG-negative higher mw band (∼300 kDa) is detectable in culture media for HEK^WT^ and HEK^SC^, but not for HEK^SC/KO:POMT1/2^ cells. In *panel A*, 10 uL each of cell lysate and cell media were loaded for WB analysis (volumetric 1:1 ratio), while in *panel B*, 10 uL cell lysate and 10 uL of 1:5 diluted cell media (v/v) was used for the WB analysis (volumetric 1:5). The reduced volume of the supernatant in *panel B* was necessary to avoid saturation of the strong secreted GFP signal and permit clear visualization of both lysate and supernatant bands on the same blot. *B*, analysis as in (*A*) with the secreted GFP-swapped KIAA1549-GFP-sol construct. Note, that the major bands in lysates corresponding to the intact protein (∼80–90 kDa) are detectable by anti-GFP, but not anti-FLAG, while a GFP-positive doublet band is detected in the culture media for all glycoengineered cells including HEK^SC/KO:POMT1/2^. *C*, Western blot analysis of Endo H and PNGase F digested cell lysates from (*A*). Note, that the *upper band* of the intact protein (160-170 kDa) is sensitive to both Endo H and PNGase F digestion, while the *lower band* is insensitive. *D*, Western blot analysis of Endo H and PNGase F digested cell lysates from (*B*). Note, that the *upper band* is sensitive to both Endo H and PNGase F digestion, while the *lower band* is insensitive.

The KIAA1549-GFP-sol construct produced a doublet band migrating at around 80-90 kDa in total cell lysates as detected by the N-terminal GFP, however there was no detection of the C-terminal FLAG tag for these bands (Fig. 4, *B* and *D*) contrary to the analysis with KIAA1549-GFP-MEM-Full (Fig. 3*B*). Moreover, a doublet band migrating higher at around 90-110 kDa was detected in the culture medium by labeling for GFP in all cells regardless of capacity for POMT1/2 O-mannosylation. This suggests that the KIAA1549-GFP-sol protein is processed and secreted regardless of POMT1/2 O-mannosylation but also suggests that the protein has undergone processing in the C-terminus to remove the FLAG tag. Furin processing of these truncated proteins is expected to result in small undetectable C-terminal fragments, but mature proteoforms accumulated in ER would be expected to retain the FLAG tag in absence of furin processing, as was observed with the KIAA1549-sol protein (Fig. 4, *A* and *C*). The discrepancy in FLAG reactivity may suggest that the KIAA1549-GFP-sol protein is efficiently trafficked to the Golgi, while for the KIAA1549-sol protein a minor fraction is still FLAG tag positive and likely ER-localized. To further investigate this, we proceeded with PNGase F and EndoH digestion, which for the KIAA1549-sol protein revealed that the upper band of the barely separated doublet band, corresponding to the intact protein (∼165 kDa), was sensitive to both enzymes, while the lower band was insensitive to both (Fig. 4*C*). This suggests that the upper band represents the ER retained N-glycosylated protein with high-Man N-glycans, while the lower band may represent a nascent protein without N-glycans. The same analysis of the KIAA1549-GFP-sol protein migrating as a doublet (∼80–93 kDa) revealed the same pattern where the upper band collapsed into the lower band with both Endo H and PNGase F (Fig. 4*D*). Thus, the N-glycan analysis appears to conflict with the interpretation that furin processing has resulted in loss of the C-terminal FLAG tag on the KIAA1549-GFP-sol protein. The reason for this has not been resolved, and we note that these truncated secreted constructs are artificial and may not reflect the full protein.

Together, these results suggest that KIAA1549-sol and KIAA1549-GFP-sol are processed differently with respect to C-terminal cleavage. In both cases, the majority of the protein carries high-mannose N-glycans and is retained in the ER, whereas only a minor fraction exits the ER and is secreted following furin-mediated processing.

### Stable expression of full-length KIAA1549 in HEK293 WT cells

**Figure 5:**
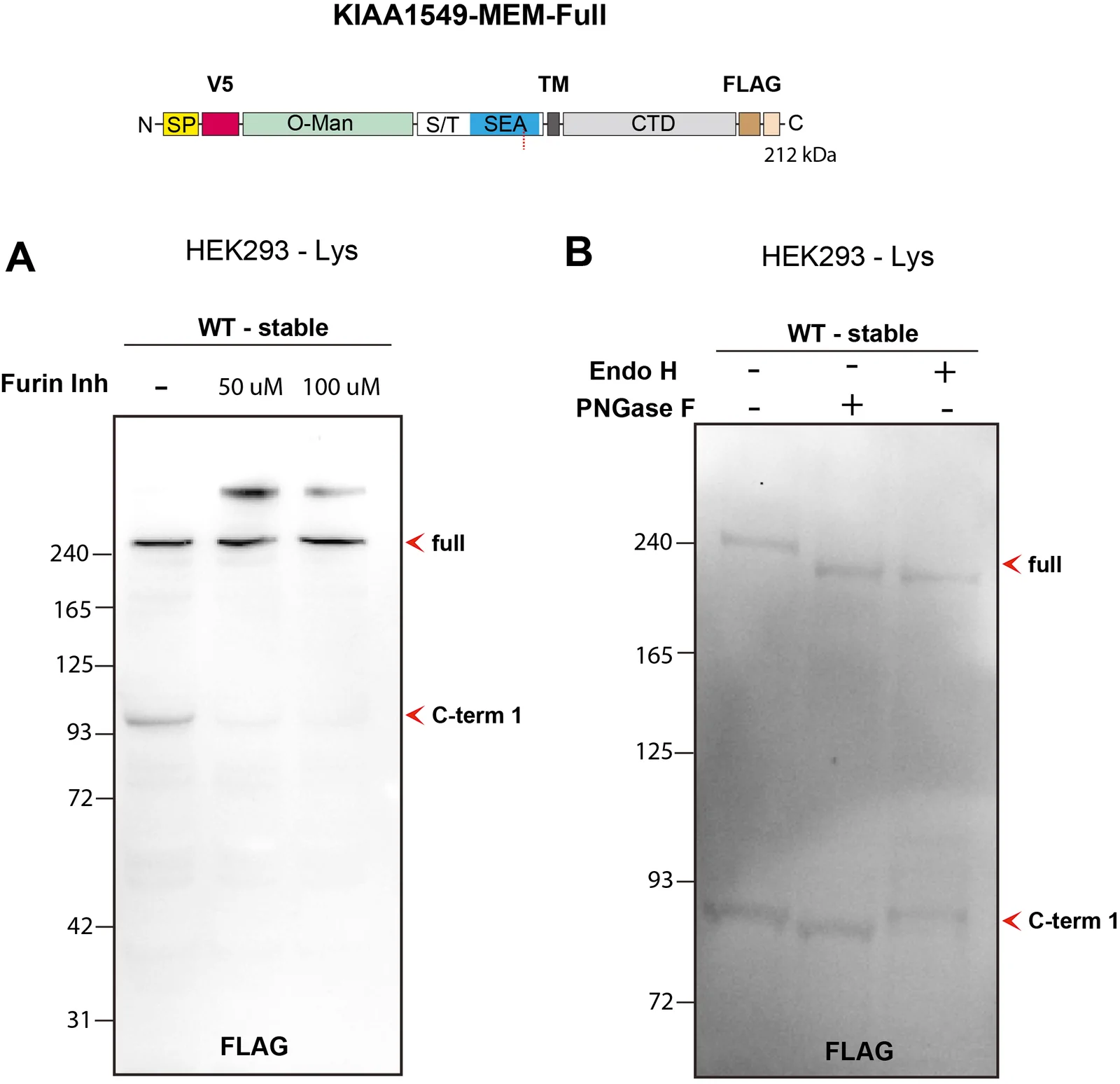
**Stable low-level expression of full-length KIAA1549 in HEK293 WT cells confirms furin processing and N-glycan maturation.***A*, Western blot analysis (anti-FLAG) of total lysates from HEK293 WT cells stably expressing KIAA1549-MEM-Full following zeocin selection and treated with the indicated concentrations of furin inhibitor I (dec-RVKR-CMK). The predicted full protein (∼240 kDa) and the C-terminal FLAG-tagged fragment (∼100 kDa) are detected at approximately equal intensities under untreated conditions. Furin inhibition resulted in disappearance of the ∼100 kDa C-terminal fragment with concomitant appearance of a slower migrating band (>300 kDa), consistent with accumulation of the uncleaved protein. *B*, Western blot analysis of Endo H and PNGase F treated total lysates from HEK293 WT cells with stable expression of KIAA1549. Note that the full protein is sensitive to both Endo H and PNGase F digestion, whereas the ∼100 kDa C-terminal fragment is resistant to Endo H but sensitive to PNGase F.

Given our findings that transient transfection resulted in apparent over expression and ER accumulation of KIAA1549, we sought to establish stable expression in HEK293 cells using zeocin selection. This was challenging and we were only able to establish stable low-level expression of the full length KIAA1549 (KIAA1549-MEM-Full) protein in HEK293^WT^ cells (Fig. 5*A*). SDS-PAGE anti-FLAG Western blot analysis of total lysates of the zeocin selected pool of cells revealed two major bands of approximately equal intensities migrating according to the predicted full protein (∼240 kDa) and the C-terminal Flag-tagged cytosolic fragment (∼100 kDa), and treatment of cells with furin inhibitor resulted in disappearance of the C-terminal fragment with concomitant appearance of a novel slower migrating band (>300 kDa), presumably representing the full protein with more complex and sialylated glycans attached following Golgi trafficking. Note that the expression level was similar to that found with viral transduction of the KIAA1549::BRAF construct (Fig. S2*A*), which was unstable upon extended culturing. Analysis of the N-glycosylation states of the full and furin-processed fragment showed that the full protein was fully sensitive to both PNGase F and Endo H, while the cytosolic fragment was insensitive to Endo H but not PNGase F (Fig. 5*B*). These results confirm the principal conclusions drawn from our studies using transient expression of KIAA1549 with respect to maturation of N-glycans following ER exit and the efficient furin cleavage of KIAA1549. Further studies with stable expression of KIAA1549 and the truncated and GFP-domain swapped constructs developed here clearly will improve insights and ultimately shed light on the fate and functions of the furin-processed N-terminal ectodomain, but these studies will have to await successful establishment of stable expression in the full panel of glycoengineered HEK293 cells.

## Discussion

Our study demonstrates how basic post-translational modifications of the KIAA1549 protein can serve supporting roles in activation of the protooncogene BRAF in the KIAA1549::BRAF fusion protein. KIAA1549 is a heavily O-mannosylated and N-glycosylated type I transmembrane protein, and our study suggests that POMT1/2 mediated O-mannosylation in the ER is necessary for transport to the Golgi, while access to the Golgi leads to furin processing and release of the C-terminal cleavage product. These post-translational properties intrinsic to the KIAA1549 protein can thus provide the KIAA1549::BRAF fusion protein with release of the cytosolic truncated BRAF protein without its inhibitory domain. These results fully recapitulate our previous findings with the KIAA1549::BRAF fusion protein and thus confirm that the KIAA1549 directly supports the activation of BRAF. We were not able to conclusively determine the fate of the furin processed N-terminal ectodomain of the KIAA1549 protein being unable to detect cell membrane expression or secretion, but our studies with transient expression of the ectodomain alone revealed evidence that the ectodomain without the TM is at least partially secreted in the presence of POMT1/2 O-mannosylation. Our study also highlights challenges with transient overexpression of KIAA1549 leading to impaired secretion and intracellular accumulation of immature proteoforms, however, we were able to establish stable expression of KIAA1549 in HEK293 WT cells and confirm that KIAA1549 undergoes efficient furin processing and acquisition of complex N-glycans.

We employed domain-swapping to evaluate the importance of the O-Man domain for expression and trafficking of KIAA1549. Replacing the O-Man domain with GFP did not appear to affect expression, trafficking to Golgi and furin processing, but clearly this substitution may influence folding kinetics, oligomerization, stability, and trafficking, which could impact the results and interpretations. Perhaps related to this, our data do not explain the differential C-terminal processing of the secretory KIAA1549-sol and KIAA1549-GFP-sol proteins. Both proteins are predicted to primarily be located in the ER based on the N-glycan analysis, but the KIAA1549-GFP-sol protein has likely undergone furin processing presumed to occur in the Golgi since the C-terminal FLAG-tag was not detectable, in striking contrast to the labeling of the KIAA1549-sol protein (Fig. 4, *B* and *D*). Further studies of the GFP-swapped KIAA1549 constructs are clearly needed, but the cumulated results of our study provide strong evidence that POMT1/2 mediated O-mannosylation is required for ER exit of KIAA1549. In contrast, the well-characterized α-dystroglycan protein appears to traffic to the Golgi independent of O-mannosylation and secrete into the culture medium, as we previously showed with an α-dystroglycan ectodomain reporter expressed in similar glycoengineered cells (9). We previously also demonstrated that in POMT1/2 KO cells, the mucin-like domain of α-dystroglycan acquires GalNAc-type O-glycosylation at sites normally occupied by O-Man glycans, which can be explained by the compartmentalization of POMT1/2 in the ER and the polypeptide GalNAc-transferases in the Golgi (15). Thus, POMT1/2 acts first, and when α-dystroglycan is expressed in cells with KO of POMT1/2, we find GalNAc-type O-glycans at sites occupied by O-Man glycans in HEK^SC^ (8). However, for KIAA1549 we have not identified similar introduction of GalNAc-type O-glycans in our previous O-glycoproteomic analyses (7, 8, 9), which is in agreement with the present finding that the KIAA1549 protein in POMT1/2 KO cells does not reach Golgi and hence is not exposed to GalNAc-transferases.

POMT1/2 are close orthologs of the large family of yeast pmt1-7 protein O-mannosyltransferases (16, 17, 18, 19), which at least partly appear to function co-translationally (20). Moreover, accumulating evidence indicates that O-mannosylation in yeast participates in ER protein quality control through interactions with chaperones and degradation machinery (21) as well as serving in termination of futile protein folding to reduce ER stress (22). The human POMT1/2 heteromeric complex differs from the yeast pmts by serving only highly select substrates (8, 9, 18), but our study suggests that POMT1/2 and their O-mannosylation functions may also serve important roles in the ER. It is well established that protein N-glycosylation serves key roles in folding, quality control, and ER to Golgi trafficking (23, 24), but other types of protein glycosylation initiated in ER also serve important roles (25). Thus, O-fucosylation of epidermal growth factor-like (EGF) repeats and thrombospondin type I repeats (TSRs) by POFUT1 and POFUT2, respectively, serve as non-canonical quality control mechanisms and are important for ER exit of the Notch receptor and members of the A Disintegrin and Metalloproteinase with ThromboSpondin Type-1 motifs (ADAMTS) family (26). More recently, the same was found for O-fucosylation of properly folded elastin microfibril interface (EMI) domains by POFUT3 and POFUT4 (27). Similarly, O-glycosylation by POGLUT1 and C-mannosylation by DPY19L1-4 are important for quality control mechanisms and secretion of their respective client substrates (28, 29). There are three types of protein O-mannosylation in animals directed by distinct ER-localized O-mannosyltransferases: POMT1/2, TMTC1-4 and TMEM260 (9, 18). While E-cadherin traffics normally in the absence of TMTC-mediated O-mannosylation (11), we previously demonstrated that TMEM260 dependent O-mannosylation of plexins, cMET and RON receptors, containing immunoglobulin, plexin and transcription factor (IPT) domains, is required for trafficking from ER to the cell surface (12). Thus, glycosylation of distinct protein folds by different O- and C-glycosylation pathways is a common feature serving in ER processing and quality control. The KIAA1549 protein stands out in this respect with its unstructured O-Man protein domain, not dissimilar to mucin-like domains (9), requiring O-mannosylation, and further studies are clearly needed to address the mechanism behind its retention in ER without the POMT1/2 enzymes. Here, we did determine that the elongation of KIAA1549 O-Man glycans is not required for trafficking by analysis of cells with KO of POMGNT1 that directs the first step in biosynthesis of elongated core M1/M2 structures.

The differential processing of KIAA1549 as a function of furin inhibition in the Golgi (Fig. 2*C*) is another interesting observation. More specifically, KIAA1549 is processed into several C-terminal fragments (C-term #1-3) in the absence of furin inhibitor, and while C-term #1 (∼100 kDa) can be explained by furin-like processing in the Golgi lumen, the formation of C-term #3 fragments is not easily accounted for since the molecular mass of these fragments can only be explained by cleavage in the CTD domain of KIAA1549 that protrudes into the cytosol, which is not accessible to furin-like proteases in the Golgi lumen. These observations tend to suggest that secondary proteolytic events, by yet unknown enzymes, occur within the CTD domain, but only when the initial furin-like cleavage of KIAA1549 has occurred in the Golgi lumen, which in part is reminiscent of the proteolytic events for the Notch receptor that undergoes ER glycosylation/folding, juxtamembrane furin-like processing within its SEA-like domain in the Golgi lumen before traffic to the cell surface where, once activated, the intracellular domain is released by a cascade of proteolytic events that allow a released C-term fragment to translocate to the nucleus and activate transcription (30, 31). Thus, the observed C-terminal #3 fragments of the KIAA1549 CTD domain may result from secondary cleavage events that indirectly depend on furin-like cleavage and maturation in the Golgi lumen, which by extension depends on the initial POMT1/2 glycosylation for ER-to-Golgi traffic. Future studies are therefore warranted to address proteolytic events within the KIAA1549 CTD, which may offer key insight into the biogenesis, maturation and functions of both KIAA1549 and oncogenic KIAA1549::BRAF fusion proteins. Specifically, it will be important to investigate whether the genetic alterations observed in pLGGs allow BRAF to access a biosynthetic route that transports BRAF to the plasma membrane *via* its fusion to the KIAA1549 protein carrier, where such secondary cleavage events upstream of the breakpoint in KIAA1549 CTD allow release of a constitutively active kinase domain. However, it should be noted that only the C-term #1 fragment was clearly detectable in the stable HEK293 WT cells expressing low levels of well secreted KIAA1549 (Fig. 5*A*).

The biological functions of the KIAA1549 protein are currently unknown, but the protein is special among O-glycoproteins with its mucin-like domain decorated by O-Man glycans rather than GalNAc-type O-glycans. The only other protein with a similar albeit shorter segment with O-Man glycosylation is α-dystroglycan, and in this protein select O-Man glycans acquire the matriglycan polysaccharides that are essential for adhesion in the Dystrophin glycoprotein complex (32, 33). The KIAA1549 protein resembles cell membrane mucins like the classical MUC1 mucin. MUC1 is a type I transmembrane protein containing an extended O-glycodomain (GalNAc-type O-glycosylation), SEA domains, a transmembrane segment, and an extended cytosolic region (34, 35). The ectodomain of KIAA1549 is also connected to the transmembrane segment through three SEA-like domains that includes the furin cleavage site in the most membrane proximal SEA domain (Fig. 1*A*). MUC1 is autocatalytically cleaved in its SEA domain in ER and remains as the full protein at the cell membrane, and its cytosolic segment engages in numerous signaling functions (36, 37). We propose that KIAA1549 similarly participates in important interactions and serve roles in signaling.

In conclusion, our study demonstrates that the KIAA1549 protein requires POMT1/2 dependent O-mannosylation to exit ER and undergo furin processing likely resulting in secretion of the ectodomain. At least in HEK293 cells the POMT1/2 O-mannosylation capacity appears to be limited and a gating factor for secretion of KIAA1549. Future studies should address the secretion of KIAA1549 and functions, and the availability of stable cell models with expression is a first step to determine this. Our results fully corroborate with our previous studies with the KIAA1549::BRAF fusion protein and highlight how KIAA1549 contributes to activation of the BRAF protooncogene.

## Experimental procedures

### Cell lines and culture conditions

Human embryonic kidney (HEK293) cells and isogenic glycosylation-deficient derivatives (POMT1/2 knockout and COSMC/POMGNT1 knockout), previously established and validated as described in (7, 8), were cultured in Dulbecco’s modified Eagle’s medium (Sigma) supplemented with 10% fetal bovine serum (Gibco) and 1% GlutaMAX (Gibco) at 37 °C in a humidified atmosphere containing 5% CO_2_. Cell lines were routinely tested for *mycoplasma* contamination.

### Plasmids and construct generation

The KIAA1549 construct C1:KIAA1549-MEM-Full was synthesized by Genewiz based on the canonical human KIAA1549 transcript (Ensembl accession ENST00000422774.2) and cloned into a mammalian expression vector (EBP67) containing an N-terminal V5 or GFP tag and a C-terminal FLAG epitope tag. The construct C2:KIAA1549-GFP-MEM-Full was generated using the same backbone, in which a region corresponding to the predicted luminal O-mannosylated segment of KIAA1549, as defined in this study, was replaced with enhanced green fluorescent protein (EGFP). The C1.3: KIAA1549-sol and C2.1: KIAA1549-GFP-sol constructs were generated from the C1 and C2 templates, respectively, using PCR-based cloning according to the In-Fusion cloning protocol (Takara Bio). The AH_p320_DelTMcyto primer pair was used to amplify and linearize the parental vectors, resulting in deletion of the transmembrane and cytosolic regions. The C1.1: KIAA1549-MEM-trunc-CTD construct was generated from the C1 template using the AH_p319_DelCTD2 primer pair to truncate the C-terminal cytosolic domain. Following PCR amplification, In-Fusion ligation was performed, and Ligation products were transformed into chemically competent *Escherichia coli* DH5α cells and plated on LB agar supplemented with kanamycin. Bacterial cultures were grown overnight at 37 °C, and plasmid DNA was isolated from individual colonies using a miniprep kit (Qiagen) prior to Sanger sequencing. Primers used for construct generation:

AH_p320_DelTMcyto_F1: 5′-AGAGCAACTCCGGAGGAGGCGACTAC-3′.

AH_p320_DelTMcyto_R1: 5′-CTCCGGAGTTGCTCTGGGACTCGGGG-3′.

AH_p319_DelCTD2_F1: 5′-TGGCCGAGTCCGGAGGAGGCGACTACAAAGACG-3′.

AH_p319_DelCTD2_R1: 5′-CTCCGGACTCGGCCACTCTGTCGGC-3′.

### Cell transfection and sample preparation

Cells were seeded at 1 × 10^6^ cells per well in 6-well plates and incubated overnight to allow attachment. The following day, culture medium was replaced with 1 ml Opti-MEM (Gibco), and cells were transiently transfected with 3 μg plasmid DNA using Lipofectamine LTX (Thermo Fisher Scientific) according to the manufacturer’s instructions. Cells were incubated for 48 h post-transfection. For analysis of total cell proteins, cells were collected by scraping and lysed in 200 μl of 1% SDS in 50 mm Tris–HCl (pH 7.5–8.0). To reduce viscosity caused by released nuclear material, lysates were treated with Benzonase nuclease (Merck) prior to further processing. Cell lysates were then prepared for SDS–PAGE and Western blotting as described below. For analysis of secreted proteins, conditioned media were collected and clarified by centrifugation at 21,000*g*. An aliquot (200 μl) of the cleared supernatant was used directly for Western blot analysis as described below.

### Stable expression of KIAA1549 under zeocin selection

HEK293 wild-type (WT) cells were seeded in 6-well plates at a density of 4.15 × 10ˆ5 cells per well and cultured in DMEM for 2 days before transfection. Cells were transfected with 1 μg of KIAA1549-MEM-Full expression construct using Lipofectamine 3000 according to the manufacturer's protocol. A stable pool of HEK293 WT cells expressing KIAA1549-MEM-Full was established by antibiotic selection with 250 μg/ml Zeocin for 3 weeks and used for the studies.

### Furin inhibitor treatment

For furin inhibition experiments, cells were seeded and transfected as described in the Cell transfection and sample preparation section. Four hours post-transfection, furin inhibitor I (Calbiochem, Merck) was added to the culture medium at a final concentration of 50-100 μm. Cells were incubated for an additional 48 h. Following treatment, cells were harvested, and cell pellets were processed for SDS–PAGE and Western blotting as described in the SDS–PAGE and Western blotting sections.

### PNGase F and endo H treatments

For enzymatic deglycosylation experiments, cells were seeded, transfected, and harvested as described in the Cell transfection and sample preparation section. Conditioned media and cell pellets were collected as above. For these experiments, cell pellets were lysed in 200 μl of lysis buffer containing 0.5% SDS, 150 mM NaCl, and 50 mm Tris (pH 8.0). Lysates were treated with Benzonase nuclease (Merck) to reduce viscosity caused by released nuclear material.

Protein denaturation and alkylation were performed by addition of dithiothreitol (DTT) to a final concentration of 10 mM, followed by incubation at 55 °C for 10 min. Samples were cooled to room temperature, after which iodoacetamide (IAA) was added to a final concentration of 25 mM and incubated for 10 min at room temperature in the dark. For deglycosylation reactions, 50 μl of each lysate was transferred to a fresh tube and supplemented with Triton X-100 to a final concentration of 1%. PNGase F digestion was performed by addition of PNGase F (5 U; New England Biolabs) followed by incubation overnight at 37 °C. For Endo H digestion, sodium acetate (pH 5.5) was added to adjust the reaction to acidic conditions, followed by addition of Endo H (New England Biolabs) and overnight incubation at 37 °C. For deglycosylation of secreted proteins, conditioned media were clarified by centrifugation as described above, and 200 μl of the supernatant was processed in parallel using the same reduction, alkylation, Triton X-100 supplementation, and PNGase F or Endo H digestion steps as described above.

Following enzymatic treatment, samples were analyzed by NuPAGE and Western blotting as described below.

### SDS–PAGE and Western blotting

Protein samples were mixed with 4× NuPAGE LDS sample buffer (Invitrogen) and reduced with 5 mM dithiothreitol (DTT) at 95 °C for 5 min. Proteins were separated by NuPAGE SDS–PAGE using precast 4–12% Bis-Tris gels (Invitrogen) run in MES running buffer. Samples treated with PNGase F or Endo H were resolved on precast NuPAGE 3–8% Tris-Acetate gels (Thermo Fisher Scientific) using Tris-Acetate running buffer. A prestained broad-range protein ladder (10-245 kDa; Abcam, ab116028) was used as a molecular weight marker. Electrophoresis was performed at 120 V for 40 min followed by 150 V for 1 h 30 min to achieve optimal protein separation. For PNGase F and Endo H treated samples, gels were run for extended times at 120 V for up to 4 h to improve resolution of glycosylation-dependent mobility shifts. For Western blotting, proteins were transferred onto 0.45 μm ethanol-activated polyvinylidene fluoride (PVDF) membranes using Tris–glycine transfer buffer (25 mM Tris, pH 8.0, 192 mM glycine, 10% ethanol). Transfers were performed at 100 V for 1 h at 4 °C. Membranes were blocked for 30 min at room temperature in blocking solution consisting of 5% skim milk in TBS-T (20 mM Tris pH 7.4, 150 mM NaCl, 0.1% Tween-20) and incubated for 1 h at room temperature with the indicated HRP-conjugated primary antibodies diluted in blocking solution: mouse anti-FLAG (M2) (Sigma, 1:4000), mouse anti-V5 (Thermo Fisher Scientific, 1:2000), or mouse anti-GFP (Thermo Fisher Scientific, 1:2000). Following three washes with TBS-T (5 min each), signals were developed using SuperSignal West Pico PLUS chemiluminescent substrate (Thermo Fisher Scientific) and imaged using an ImageQuant LAS 4000 system (GE Healthcare). Image analysis and densitometric quantification of Western blot bands were performed using Fiji (ImageJ) software. Processing efficiency was calculated as the ratio of the processed band intensity to the total signal (processed plus unprocessed bands) for each sample. Western blot analyses and quantifications were performed from three independent biological replicates.

## Data availability

All data described in this study are contained within the manuscript or supplementary figures and tables.

## Supporting information

This article contains supporting information.

## Conflict of interest

The authors declare the following financial interests/personal relationships which may be considered as potential competing interests:

P. B. has received grant funding from the Novartis Institute of Biomedical Research and has served on paid advisory boards for QED Therapeutics and Day One Biopharmaceuticals. R.B. consults for and owns equity in Scorpion Therapeutics, Karyoverse Therapeutics, and LOH Therapeutics. S. A. M. consults for and owns equity in Karyoverse Therapeutics and LOH Therapeutics. All other authors declare that they have no competing interests.
