# Supplementary material for: O-mannosylation and furin processing are critical maturation events for the KIAA1549 protein implicated in pediatric pilocytic astrocytomas
Source: J Biol Chem. 2026 Aug 7;302(10):113403. doi: 10.1016/j.jbc.2026.113403 (PMC13570299; doi:10.1016/j.jbc.2026.113403)
Supplement: Supplementary file 1 [file mmc1.pdf]

Supplementary figure 1

KIAA1549 — Human (7q34)
UniProt: Q9H5J8 | NCBI Gene: 57670 | RefSeq: NP_056061.3

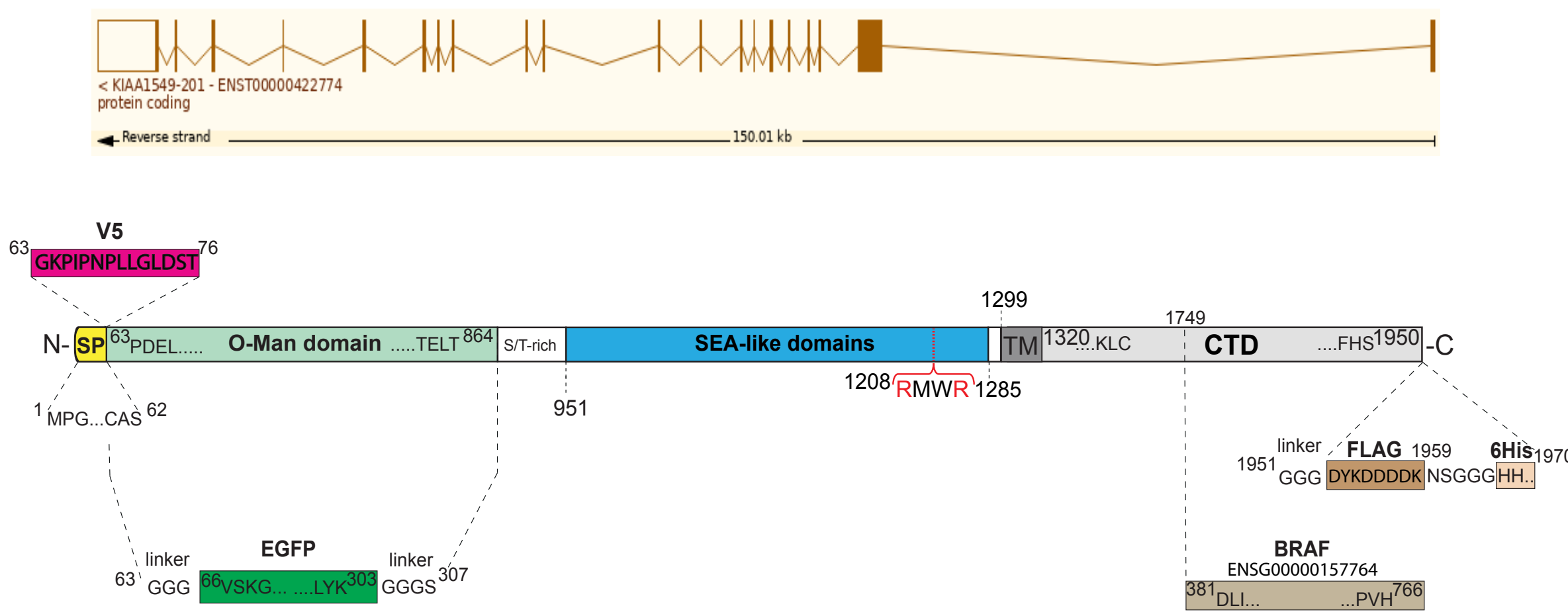

Supplementary figure 1. Domain organization of KIAA1549 and design of KIAA1549 expression constructs.

Schematic representation of the human **KIAA1549** protein and the expression constructs used in this study. The full-length KIAA1549 sequence is shown based on database annotations (UniProt Q9H5J8; NCBI Gene 57670; RefSeq NP_056061.3), with domain boundaries and key sequence features indicated. KIAA1549 is a type I transmembrane protein comprising an N-terminal signal peptide (SP), an extended O-mannosylated ectodomain (O-Man domain), three SEA-like domains, a single transmembrane (TM) segment, and a cytosolic C-terminal domain (CTD). The predicted furin-like cleavage site (RMWR) within the juxtamembrane SEA-like region is indicated.

For the **KIAA1549-MEM-Full** construct, an N-terminal V5 epitope tag was inserted between the signal peptide and the O-Man domain at the indicated amino acid positions. At the C-terminus, FLAG and 6×His tags were added sequentially to the end of the KIAA1549 coding sequence. All epitope tags were introduced using short linker sequences composed of glycine residues or glycine/serine combinations to minimize structural interference.

The **KIAA1549-GFP-MEM-Full** construct was generated by replacing the O-Man domain (aa 72–864) with enhanced green fluorescent protein (EGFP). In this construct, the signal peptide is followed directly by EGFP, which also serves as an N-terminal detection tag in immunoblot analyses; the V5 tag is not present. The GFP module was inserted using short glycine-based

linker sequences, and the C-terminal FLAG and 6×His tags were retained as in KIAA1549-MEM-Full.

Additional constructs used in this study represent C-terminally truncated or secreted versions derived from KIAA1549-MEM-Full or KIAA1549-GFP-MEM-Full and retain the same N- and/or C-terminal epitope tags as indicated.

The **KIAA1549::BRAF (15/9) fusion** included in this study incorporates the BRAF kinase domain derived from human BRAF (Ensembl ENSG00000157764). In this fusion, BRAF is truncated N-terminally at amino acid 381, thereby removing the regulatory CR1 and CR2 domains, while retaining the kinase domain spanning residues 381–766, which is fused in-frame to KIAA1549.

Supplementary figure 2

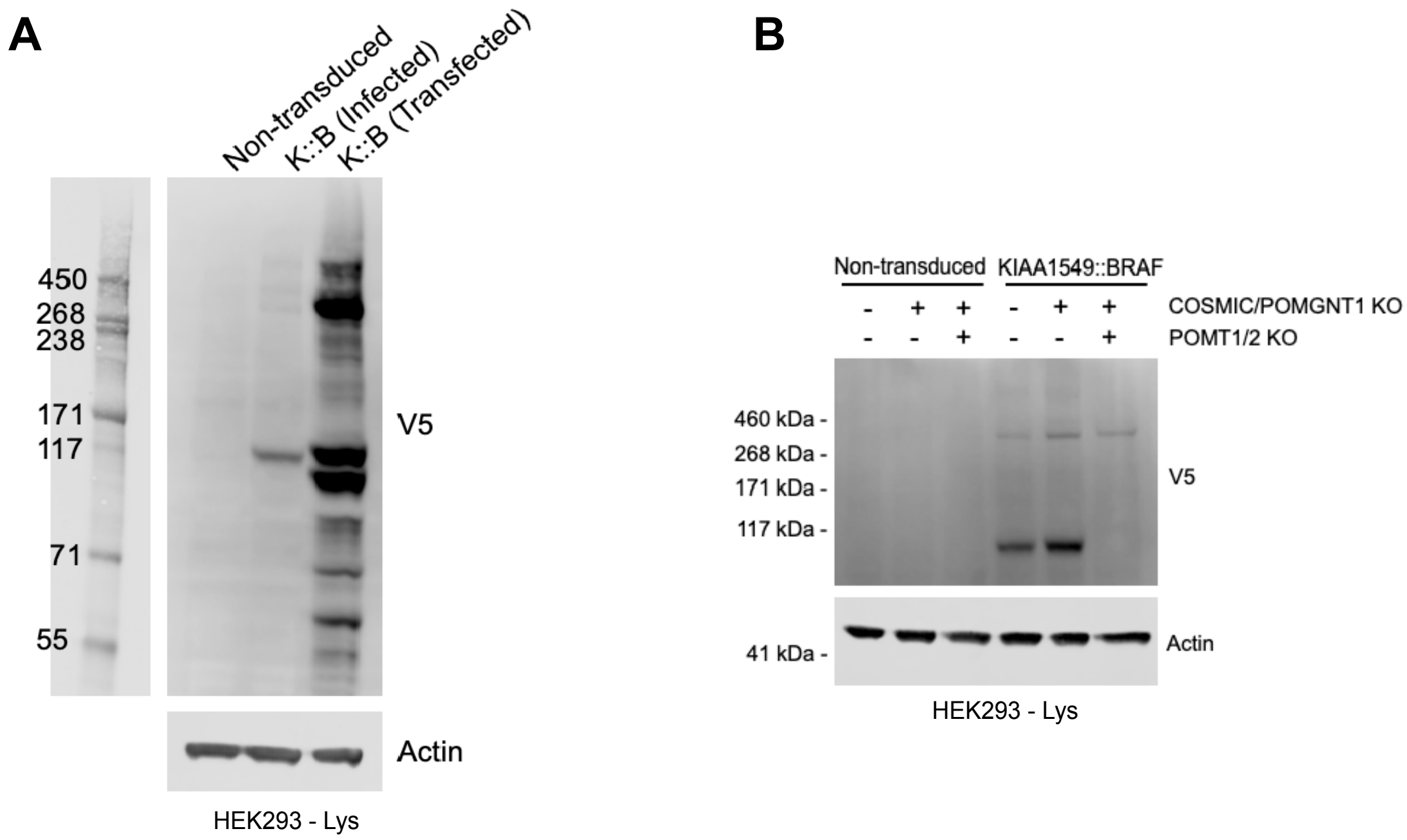

Supplementary figure 2. Differential processing of KIAA1549::BRAF following transient transfection versus stable viral transduction.

(A) Western blot analysis of total cell lysates from HEK293 cells expressing the KIAA1549::BRAF fusion protein either by transient plasmid transfection or by stable viral transduction followed by puromycin selection (generated by collaborators in Boston). Lysates were analyzed using an anti-V5 antibody to detect KIAA1549::BRAF and anti-actin as a loading control. Transient transfection resulted in prominent accumulation of the full-length fusion protein together with lower levels of the cleaved fragment, consistent with ER retention and limited Golgi processing. In contrast, stable viral transduction yielded predominantly the cleaved KIAA1549::BRAF species, with only minimal detectable full-length protein, indicating efficient trafficking to the Golgi and proteolytic processing.

(B) Replication of KIAA1549::BRAF processing following viral transduction in glycoengineered HEK293 cell backgrounds. Cells were transduced with KIAA1549::BRAF and analyzed by Western blotting using anti-V5 and anti-actin antibodies. In agreement with the results in panel A, viral transduction produced a pattern characterized by robust accumulation of the cleaved fragment and markedly reduced levels of the full-length fusion protein. This contrasts with the

processing pattern observed following transient transfection, where higher expression levels correlate with increased retention of full-length KIAA1549::BRAF and reduced cleavage.

Supplementary figure 3.

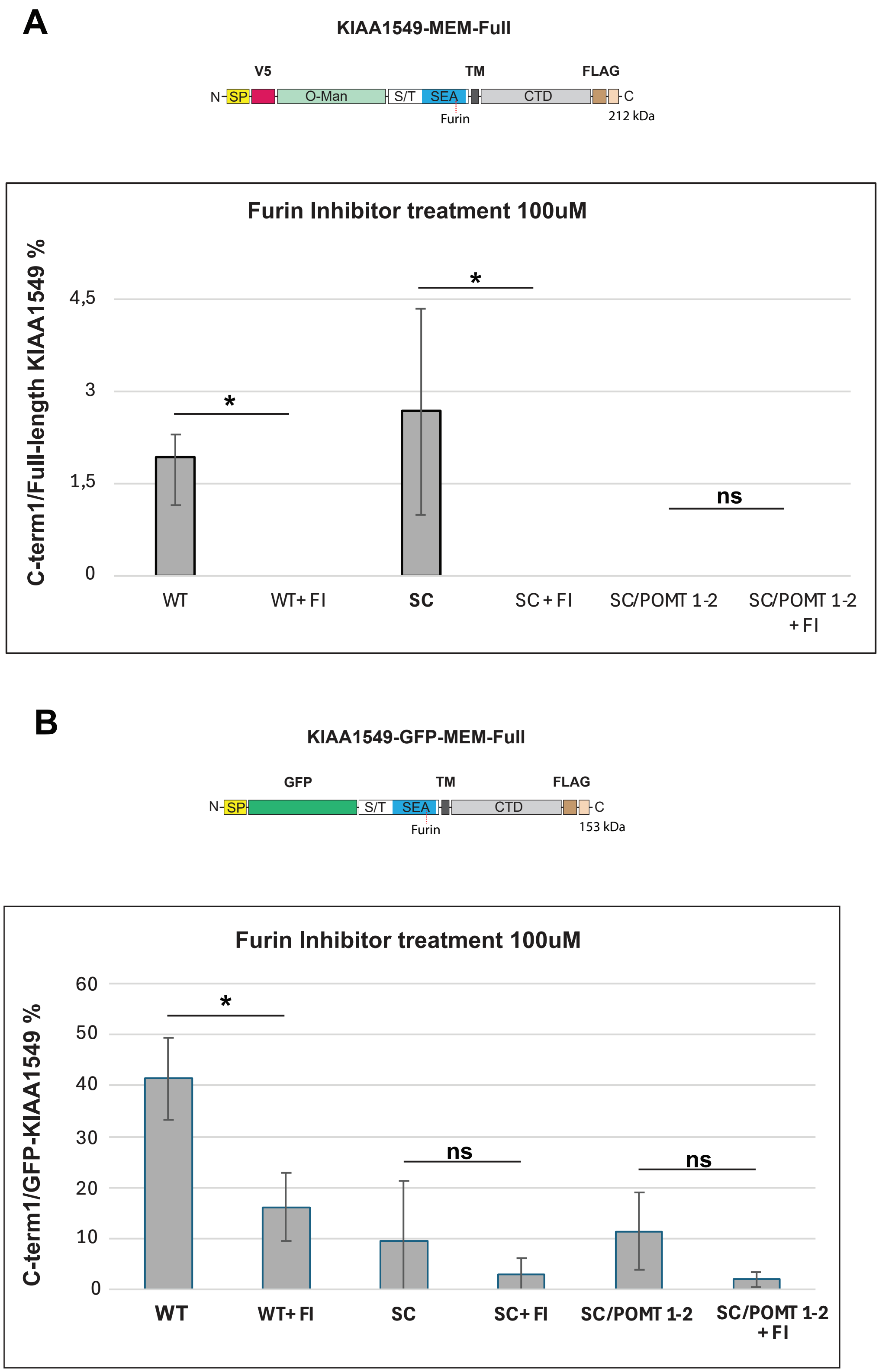

Supplementary Figure 3. Quantification of the furin-processed C-terminal fragment of KIAA1549.
A. Densitometric quantification of the C-term 1/full-length ratio for the KIAA1549-MEM-Full construct following treatment with 100 µM furin inhibitor I. **B.** Densitometric quantification of the C-term1/full-length ratio for the KIAA1549-GFP-MEM-Full construct following treatment with 100 µM furin inhibitor I.The intensity of C-term 1 was normalized to the corresponding full-length protein within the same lane. Bars represent the mean ± SD from three independent experiments (n = 3). Statistical significance is indicated in the graphs (P < 0.05; P < 0.01; P < 0.001; ns, not significant).
